# Geometric metasurface enabling polarization independent beam splitting

**DOI:** 10.1038/s41598-018-27876-2

**Published:** 2018-06-21

**Authors:** Gwanho Yoon, Dasol Lee, Ki Tae Nam, Junsuk Rho

**Affiliations:** 1 Department of Mechanical Engineering, Pohang University of Science and Technology (POSTECH), Pohang, 37673 Republic of Korea; 2 Department of Materials Science and Engineering, Seoul National University, Seoul, 08826 Republic of Korea; 3 Department of Chemical Engineering, Pohang University of Science and Technology (POSTECH), Pohang, 37673 Republic of Korea; 4 National Institute of Nanomaterials Technology (NINT), Pohang, 37673 Republic of Korea

## Abstract

A polarization independent holographic beam splitter that generates equal-intensity beams based on geometric metasurface is demonstrated. Although conventional geometric metasurfaces have the advantages of working over a broad frequency range and having intuitive design principles, geometric metasurfaces have the limitation that they only work for circular polarization. In this work, Fourier holography is used to overcome this limitation. A perfect overlap resulting from the origin-symmetry of the encoded image enables polarization independent operation of geometric metasurfaces. The designed metasurface beam splitter is experimentally demonstrated by using hydrogenated amorphous silicon, and the device performs consistent beam splitting regardless of incident polarizations as well as wavelengths. Our device can be applied to generate equal-intensity beams for entangled photon light sources in quantum optics, and the design approach provides a way to develop ultra-thin broadband polarization independent components for modern optics.

## Introduction

Metasurfaces composed of miniaturized antenna arrays provide an approach to flat optics that uses ultrathin optical components instead of conventional bulky components. A primary challenge of metasurfaces is to design a system that converts a certain incident optical wave to a desired output, and most effort focuses on wave-front manipulation due to its versatile applications such as beam steering devices^1,2^, achromatic lenses^3–6^, optical skin cloaks^7^, holographic devices^8–11^, and others^12–15^. In addition, the light controllability of metasurfaces can be further improved by combining them with dynamically-tunable mechanisms^16–21^ or nonlinear optical effects^22–25^.

Most metasurfaces operate for linearly or circularly polarized light. The antenna design principle for the linear polarization relies on resonance tuning of each antenna by controlling its physical shape. V-shaped and elliptical antennas can be used for this purpose^1,26^. The polarization-dependence can be removed by using a circular antenna rather than the elliptical one^27–29^. Circular antennas can operate for any kind of polarization, but they are highly resonant, so metasurfaces composed of circular antennas have only narrow working frequencies. Geometric metasurfaces (GEMs) that operate for the circular polarization have a broad range of working frequency because they generate phase delay by rotating the antennas, which is less resonant than the resonance tuning method. If we consider each rotating antenna as a one-dimensional dipole oscillator, the rotation angles of the antennas impart an initial phase difference to outgoing circularly polarized light. The outgoing wave from the GEM can be decomposed into co-polarized and cross-polarized light. Co-polarized beam retains its original phase distribution, so the propagation of the co-polarized beam does not change, i.e. it produces a zeroth-order beam. Only the cross-polarized beam follows the phase distribution of the metasurface. GEMs have an intuitive design principle so that we can easily determine phase variance by choosing an appropriate rotation angle for each antenna.

A beam splitter is an optical component that can divide one incident light into several beams, and there are many ways to realize such component. Typically, we can make cube-shaped beam splitters by attaching two trigonal prisms, and its functionality can be controlled by changing properties of the interface. In contrast to the conventional beam splitters, metasurfaces can serve similar functions with ultra-thin thickness by managing specific designs of metasurfaces. Typical metasurface beam splitters have a linear phase distribution consist of unit structures which cause opposite phase variation for orthogonal linear or circular polarizations^30–34^. Therefore, incident beam can be separated after passing through the metasurface as in typical Wollaston prisms. In this case, power ratio of diffracted beams is not identical, i.e. it depends on the incident polarization state. The Rochon prism which leaves ordinary ray undeviated can also be realized by controlling both propagation and geometric phase of each unit structure^35^, but its working frequency is strictly limited to a narrow band.

In modern optics such as quantum communication, equal-intensity coherent beams are usually required to generate entangled photon light sources^36–39^. Some of related works on metasurfaces to generate equal-intensity beams at NIR wavelengths regardless of incident polarization are reported^40,41^, but their working frequencies are limited to NIR region. In addition, the number of generated beams is not controllable, i.e. they always generate two diffracted beams. By attaching two equivalent metasurfaces with opposite linear phase gradient, equal-intensity beams can also be generated^42^. However, its functionality is highly affected by alignment of light source and the metasurface.

Here we propose and experimentally demonstrate GEM-based holographic beam splitter that always generates equal-intensity beams regardless of incident polarization at visible wavelengths. In principle, the number of generated beams which have equal-intensity from our device is controllable. Our device achieves both broadband characteristics and the polarization-independence by using Fourier holography on GEMs. The term of ‘broadband’ in this work does not mean consistent efficiency. Instead, it means that our device functionality can be maintained in a certain wavelength range because phase distribution of our metasurface does not change by incident wavelengths. We notice that a reversed conjugate image of Fourier hologram appears at the same image plane as the original image^43^. If the original image has origin-symmetry, the original and conjugate images can overlap exactly. Right circular polarization (RCP) and left circular polarization (LCP) generate the conjugate images each other from GEMs encoded by Fourier hologram, so we can realize GEMs that produce exactly the same outputs in any kind of polarization states by encoding an origin-symmetric image. The proposed device is experimentally demonstrated using hydrogenated amorphous silicon (a-Si:H), and diffracted beam power of the outgoing optical wave is symmetric regardless of polarization. The efficiency of our device can be further improved by using low loss materials in visible region such as crystalline silicon (c-Si)^44–46^, titanium dioxide (TiO_2_)^47,48^ and gallium nitride (GaN)^49–51^ without changing our fundamental design principle. Our device and design approach can be applied to interferometry in classical optics as well as generation of entangled photon light sources in quantum optics.

## Results

**Figure 1:**
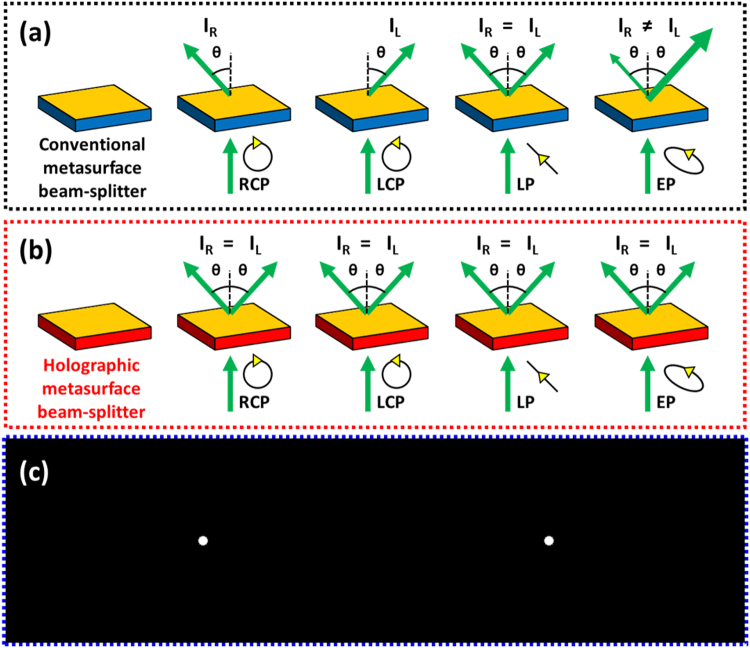
Operation schematic of the holographic metasurface beam splitter compared to the conventional one based on linear phase gradient. (**a**) The GEM with linear phase gradient does not maintain consistent functionality on different incident polarizations because each antenna produces opposite phase delays against RCP and LCP. (**b**) The proposed holographic beam splitter functions identically under any kind of polarization. The responses of the device to RCP and LCP are exactly the same. Since all polarization states can be decomposed into a sum of RCP and LCP, the device functionality is not affected by polarization. (**c**) Encoded image in the hologram contains two white spots on a black background. The image is origin-symmetric, so it can overlap perfectly with its conjugate image. This symmetry enables polarization independent operation of our device. The number of generated beams is not limited to two, and varies depending on what the image is encoded.

Our device belongs to the GEM; nevertheless, it exhibits consistent functionality regardless of the incident polarization (Fig. 1a,b). Conventional beam splitters based on the GEM has been designed by linear phase gradient which leads to separation depending on the circular polarization direction^31,32,34^. Rotating antennas of GEMs generate opposite phase delays against a pair of orthogonal circular polarizations, i.e. RCP and LCP, so the optical response of the conventional GEM varies depending on the incident polarizations. Therefore, the power ratio between diffraction beams varies depending on the incident polarization states. To remove the polarization-dependence, Fourier holography is employed to exploit the conjugate functionality of RCP and LCP. The conjugate image of Fourier hologram appears in reverse at the same position as the original image. Nanoantennas based on the geometric phase generate opposite phase variation against RCP and LCP. When circularly polarized plane wave is incident to the GEM, complex amplitudes of transmitted cross-polarized light which are RCP and LCP immediately after the metasurface are given by

1 $${\Psi }_{RCP}=A(x,y){e}^{j\varphi (x,y)},$$

2 $${\Psi }_{LCP}=A(x,y){e}^{-j\varphi (x,y)},$$

where *x* and *y* represent the coordinate of the metasurface. *A*(*x, y*) is the amplitude of the transmitted cross-polarized light, and *ϕ*(*x, y*) indicates the phase distribution of the metasurface. Amplitude variation by the metasurface can be ignored because all the nanoantennas in it are designed to induce the same amplitude change. The only difference between the complex amplitude of RCP and LCP is the sign of the imaginary part, so they have a conjugate relation as

3 $${\Psi }_{RCP}={\Psi }_{LCP}^{\ast }.$$

Intensity distributions at the image plane can be expressed as

4 $${I}_{RCP}(x^{\prime} ,y^{\prime} )\propto {[F\{{\Psi }_{RCP}\}]}^{2},$$

5 $${I}_{LCP}(x^{\prime} ,y^{\prime} )\propto {[F\{{\Psi }_{LCP}\}]}^{2},$$

where *x′* and *y′* indicate the coordinate at the image plane, and *F*{*∙*} denotes the two-dimensional Fourier transformation operator. The conjugate symmetry of the Fourier transformation yields

6 $${F\{{\Psi }_{RCP}\}]}_{(x^{\prime} ,y^{\prime} )}={F\{{\Psi }_{LCP}\}]}_{(-x^{\prime} ,-y^{\prime} )}.$$

Finally, the intensity distributions of RCP and LCP are related as

7 $${I}_{RCP}(x^{\prime} ,y^{\prime} )={I}_{LCP}(-x^{\prime} ,-y^{\prime} ).$$

Therefore, if the encoded image is origin-symmetric, the generated images by RCP and LCP exactly overlap each other.

The origin-symmetric image of two white spots with a black background is encoded in our metasurface based on computer-generated phase-only Fourier holography (Fig. 1c). The original image has a size of 800 × 200 pixels, which is expanded to 1250 × 1250 pixels by using zero padding to increase the image fidelity. We use the Gerchberg-Saxton (GS) algorithm^52^ to derive a desired phase distribution from the enlarged image by two hundred iterative processes combined with the initial random phase mask. The random phase mask is employed to the original image for easy convergence of the algorithm. It is usually attached to the object image to spread the energy over a wide spectrum to ignore the amplitude of a complex Fourier hologram^53^. Otherwise, most of the energy concentrates in the zeroth order beam and the effective dynamic range is reduced. The Fourier hologram can be represented in the complex form of *H*(*x, y*) = *A*(*x, y*)*exp*[*−jφ*(*x, y*)]. If we can ignore the amplitude *A*(*x, y*), a gray-tone image can be generated by controlling *φ*(*x, y*) only, which refers to the phase-only hologram. The derived phase distribution is discretized into eight uniform phase steps from 0 to 2*π*. The arrangement of the metasurface elements according to the hologram corresponds to the discrete phase distribution from the GS algorithm. Eight uniform phase steps match eight uniform rotation steps of nanoantennas from 0 to *π* in our metasurface.

The phase profile of our metasurface is not equivalent of two diffraction grating placed next to each other. The phase profile retrieved from the GS algorithm is not origin symmetric because the random phase mask is attached to the original origin symmetric image of two spots. The value of each pixel of the image with the random phase mask becomes complex number which is no more origin symmetric. The phase profile of our metasurface looks linear, but one can easily notice that it is not exactly linear (Supplementary Fig. S1). Orientation of the nanorods varies not only horizontally, but also vertically. The vertical variation results from the GS algorithm with the random phase mask. If our device has origin symmetric phase profile, the orientation of the nanorods should not be changed in vertical direction.

The Fourier hologram uses a Fourier-transformed image to encode the hologram, so the outgoing optical wave from the hologram should be Fourier transformed again to recover the original image. A convenient way to conduct Fourier transformation of optical waves is to let the wave propagate to an infinitely-distant plane; this process works the same as a thin optical lens. Although an infinitely-distant plane does not exist in the real world, our metasurface is small enough, i.e. 300 μm × 300 μm, so an image plane a few centimeters away from the metasurface can be regarded as infinitely distant without causing significant error. The generated holographic image only experiences magnification without change of overall configuration along the distance from the hologram consistently maintaining two circular spots; hence, it works as a beam splitter generating equal-intensity beams.

The most attractive advantage of our device is that it can generate two or more equal-intensity beams simply by changing the encoded image. In this work, we use two point image with origin symmetry for the hologram encoding. If we use four point image with origin symmetry, we can obtain four equal-intensity beams. Theoretically, any even numbers of equal-intensity beams are possible while odd numbers are impossible due to the origin symmetry condition. The metasurface beam splitters based on one dimensional binary phase profile can also increase the number of beams by arranging two dimensional binary phase profile, but controllability is not as high as our approach because our device can use eight or more phase steps.

**Figure 2:**
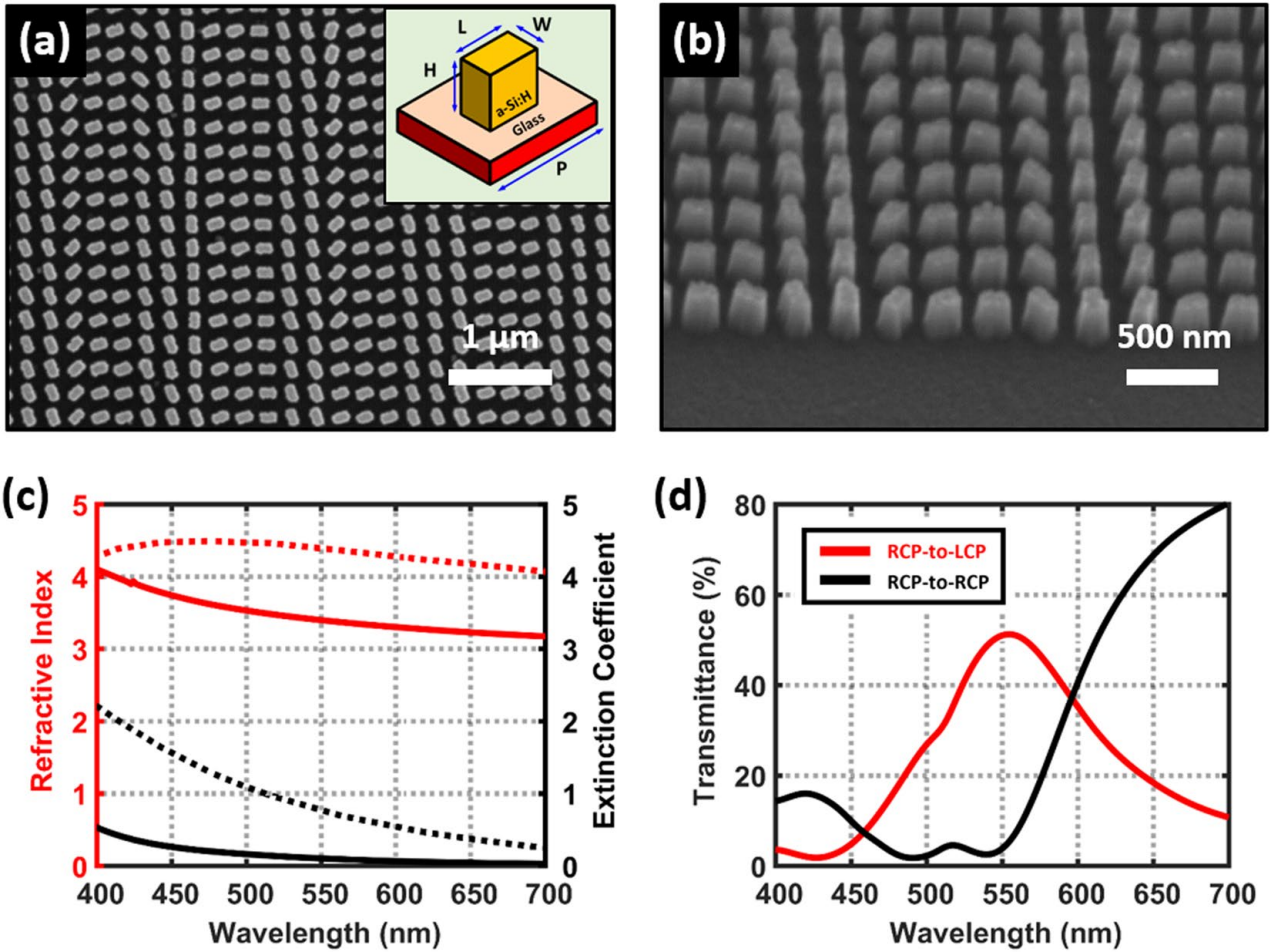
Fabrication of the metasurface and its optical properties. (**a**,**b**) SEM image of the fabricated metasurface in the top and tilted view. The phase distribution is different in our metasurface than in the conventional metasurface beam splitter which has a one-dimensional linear phase distribution. The a-Si:H is used as the structuring material to cover visible working frequencies. inset: Geometry of the unit cell: length *L* = 150 nm, width *W* = 80 nm, height *H* = 300 nm, pitch *P* = 240 nm. (**c**) Comparison of the measured complex refractive indices of our a-Si:H (solid lines) and the literature value of unhydrogenated amorphous silicon^58^ (dotted lines). Red lines represent the refractive index while black lines show the extinction coefficient for both materials. Hydrogen impurities decrease the extinction coefficient of amorphous silicon in visible wavelengths improving the CPT. (**d**) The calculated transmittance spectrum of the designed unit cell. The red line represents cross-polarization while the black line shows co-polarization. The transmittance in shorter wavelength region can be improved by using low loss materials such as c-Si, TiO_2_ and GaN.

The a-Si:H as structuring material for our metasurface achieves a broadband functionality in visible wavelengths (Fig. 2a,b). The a-Si:H refers to amorphous silicon which has hydrogen impurities. The impurities decrease the defect density of amorphous silicon, so a-Si:H has a much lower extinction coefficient than typical amorphous silicon in visible wavelengths^54^ (Fig. 2c). We also calculate the cross-polarization transmittance (CPT) of the unit cell made of the a-Si:H (Fig. 2d). The CPT is defined as the ratio of the power of the transmitted cross-polarized light to the total incident optical power, and the calculated CPT reaches up to 50% at the wavelength *λ* = 550 nm. The size and pitch of nanoantennas are determined based on the calculated CPT. The conversion efficiency increases as the CPT increases, so they are designed to maximize the CPT minimizing the reflectance (Supplementary Fig. S2). If we exploit other materials such as c-Si, TiO_2_ and GaN whose extinction coefficient is lower than a-Si:H, the CPT of the metasurface can be further improved.

**Figure 3:**
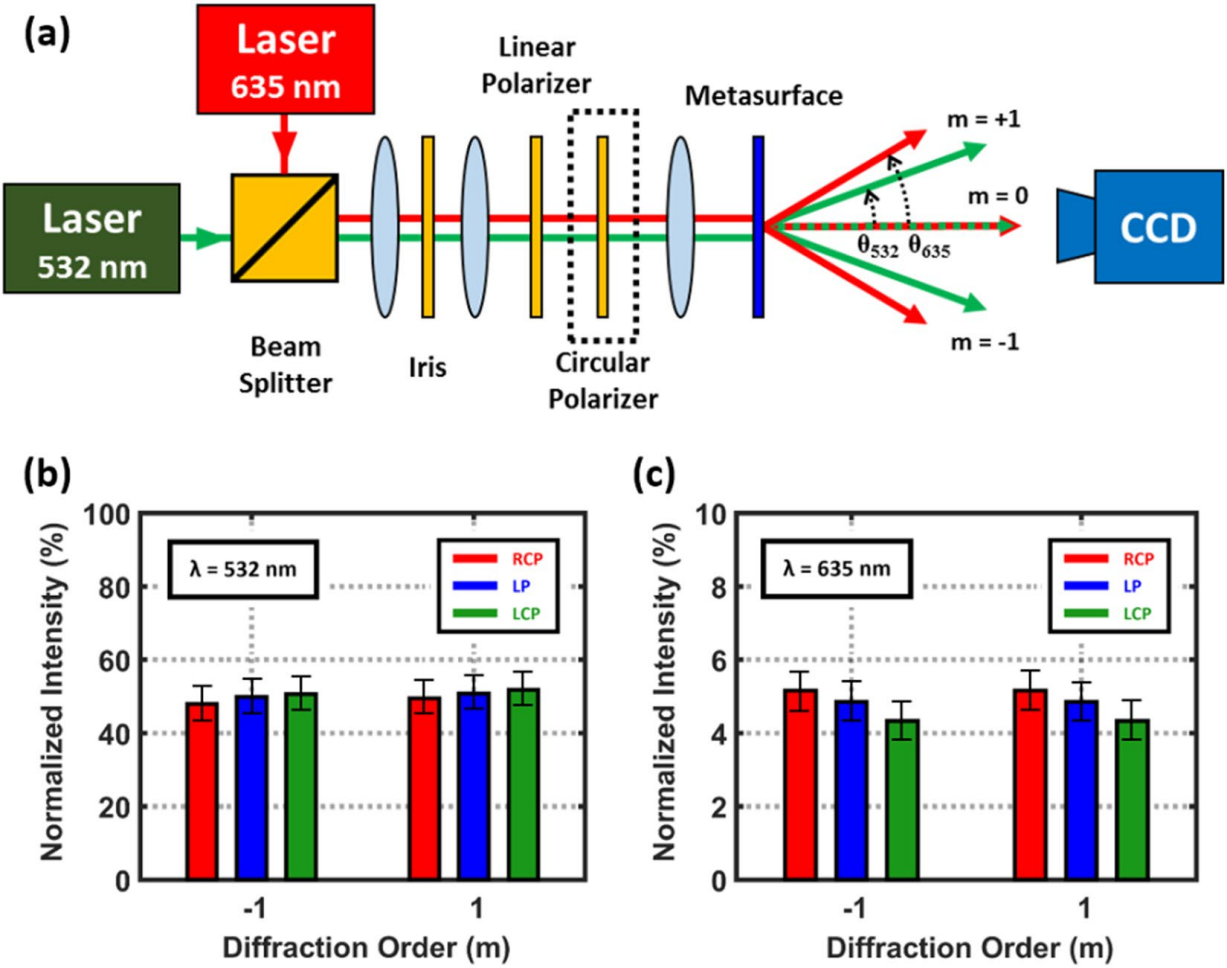
Characterization of diffraction power and illustration of measurement setup. (**a**) The optical setup to characterize our metasurface beam splitter. Diffraction angles vary depending on the incident wavelength, i.e. *θ*_532_ ≈ 24° and *θ*_635_ ≈ 28.5°. (**b**,**c**) Measured beam powers by diffraction orders, incident polarizations and wavelengths. Diffraction orders of *m* = +1 and *m* = −1 correspond to the left and right spot of the encoded image, respectively. All measured powers are normalized to each zeroth-order power.

**Figure 4:**
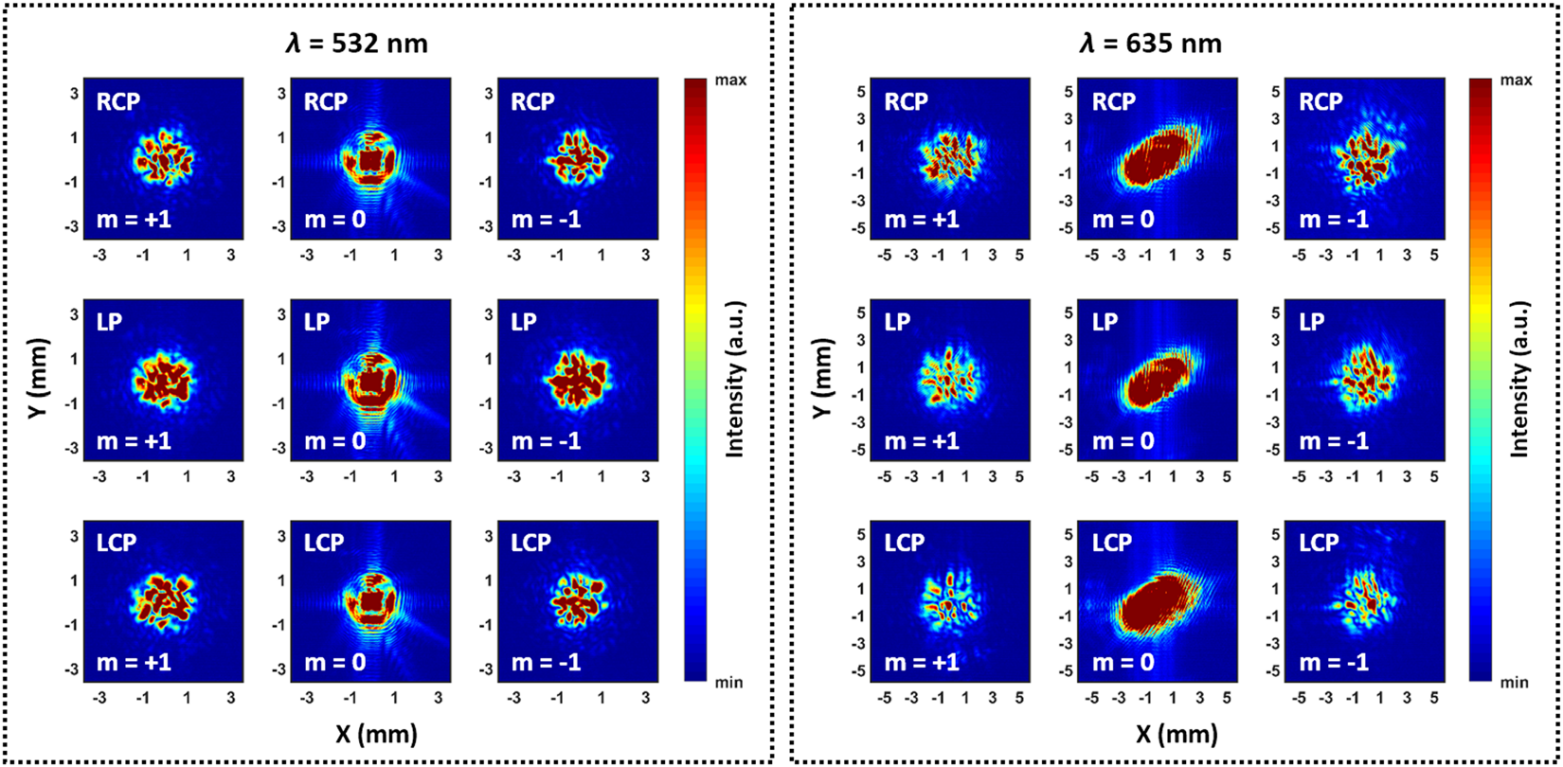
Measured optical intensity distributions at the image plane. LP denotes a linear polarization state. Diffracted beams have random distributions because of laser speckles, but are located within a certain boundary that can be regarded as the beam size. The image plane is located 14 cm behind the metasurface, and the beam diameters can be estimated as ~3 mm for *λ* = 532 nm and ~5 mm for *λ* = 635 nm. Corresponding beam diverging angles are calculated from the estimated beam diameters as 2.5° and 4.1°, respectively. Zeroth-order distributions show that incident beam sizes are bigger than our metasurface, so some of incident light go directly to the laser power meter without any interaction with the metasurface. Therefore, the measured diffraction efficiencies become lower than the simulation results.

The fabricated metasurface shows the consistent beam-splitting regardless of the incident polarizations (Fig. 3). The diffraction angle increases as incident *λ* increases, but is not affected by the incident polarization. The powers of diffracted beams are characterized by diffraction orders, incident polarizations and wavelengths. As a result, the power of each diffraction order of *m* = +1 and *m* = −1 is always equal for RCP and LCP as well as wavelengths. Arbitrary polarization states can be decomposed into the combination of RCP and LCP, so our device functionality is valid for the arbitrary polarization states. The normalized intensity difference is originated from the instability of the laser power; nevertheless, measured values by each polarization can be supposed to be equal within the error range. Total efficiency which is defined by the ratio of the sum of diffraction beam powers (*m* = +1, −1) to the incident beam power is 18.3% at *λ* = 532 nm and 9.1% at *λ* = 635 nm. This difference agrees with the fact that the CPT is higher at *λ* = 532 nm than at *λ* = 635 nm wavelength. Diffraction efficiency which is defined by the ratio of the sum of diffraction beam powers to the total transmitted beam power at *λ* = 532 nm is 10 times higher than at *λ* = 635 nm. The difference of the diffraction efficiency mainly comes from the incident beam size and shape. In the case of 635 nm, we use a laser diode which has elliptical beam shape (Fig. 4). If we use this source to cover the square shaped metasurface, considerable amount of incident light does not pass the metasurface resulting diffraction efficiency decrease. It causes the difference as well as the discrepancy with the simulation. However, it does not affect our device functionality because our device still generates same powered diffraction beams.

The intensity distributions of each diffracted beam are also measured using a charge-coupled device (CCD) (Fig. 4). Regarding of the saturation, a power meter is used to measure the beam powers while the CCD is used to capture the intensity profiles. The CCD is saturated to clearly show the beam profiles and sizes, but the power meter is not saturated because the incident beam power is less than the measurement limit of the power meter. Optical power is limited to a certain boundary, but randomly distributed due to the appearance of laser speckles. The strong coherence of the laser source causes the speckles, and this disturbance can be reduced using a diffuser^48^ or Dammann grating^9,55^.

The broadband polarization independent beam splitting can be realized by using circular pillar based metasurfaces which does not belong to the GEM^56^. However, our approach as a GEM has advantages of robustness to the fabrication error as well as intuitive design process to achieve broadband functionality. Phase variation from the circular pillar based metasurfaces is strongly affected by the size of structures which always has discrepancy with the design due to the perturbation in fabrication conditions. In contrast, phase variation from the GEM is determined by the orientation of the nanorods which is less sensitive to the fabrication error. This is a general advantage of GEMs. In addition, by using GEM we can achieve broadband characteristics without any further algorithm.

In summary, we investigate and experimentally demonstrate a holographic beam splitter that generates equal-intensity beams with incidence of any polarization at visible wavelengths. In contrast to conventional metasurface beam splitters of linear phase gradient, Fourier holography is exploited to maintain the consistent functionality for incident polarization. A perfect overlap of an original and a conjugate image of Fourier hologram is a key concept of our device principle. Theoretically, the number of the generated beams can be controlled by replacing the two point image into others, e.g. four point and eight point images. The fabricated metasurface based on a-Si:H performs consistent beam splitting regardless of incident polarizations as well as wavelengths, and the efficiency can be improved by using low loss materials. Our device can be exploited to generate two or more entangled photon light sources in quantum optics, and design approach provides a platform for broadband polarization independent optical components of modern optics.

## Methods

### Sample Fabrication

Plasma-enhanced chemical vapor deposition (HiDep-SC, BMR Technology) was used for deposition of 300-nm-thick a-Si:H thin film on a fused silica substrate. Deposition rate was 1.3 nm/s under the temperature of 300 °C with 10 sccm flow rate of SiH_4_ gas and 75 sccm flow rate of H_2_ gas. A chromium (Cr) hard mask was defined on the a-Si:H by using conventional electron beam lithography (ELS-7800, Elionix) with electron beam evaporation (KVE-E4000, Korea Vacuum Tech). Inductively-coupled plasma reactive ion etching was employed to etch the a-Si:H film along the Cr mask. 80 sccm flow rate of Cl_2_ gas and 120 sccm flow rate of HBr gas were used as etching gas resulting the etching speed of 4 nm/s. The remaining mask was removed using Cr etchant.

### Material Characterization

The a-Si:H thin film was characterized using ellipsometry (M-2000, J.A. Woollam). The incident beam angle was 65°. The Tauc-Lorentz model developed by Jellison and Modine was employed because this model generally works well with the absorptive amorphous material^57^. The imaginary part of the dielectric function of the Tauc-Lorentz model is given by

$$\varepsilon ^{\prime\prime} (E)=\begin{array}{cc}\frac{A{E}_{0}B{(E-{E}_{G})}^{2}}{E[{({E}^{2}-{E}_{0}^{2})}^{2}+{B}^{2}{E}^{2}]} & when\,E > {E}_{G}\\ 0 & when\,E\le {E}_{G}\end{array}$$

where *A* is the strength of the absorption peak, *B* is the broadening term, *E*_0_ is the energy position of the absorption peak, and *E*_*G*_ is the optical band gap energy. The fitting parameters for our material were *A* = 93.974 eV, *B* = 2.339 eV, *E*_0_ = 4.128 eV, and *E*_*G*_ = 1.271 eV. The mean square error between the fitting curve and the measured data was 11.123.

## Electronic supplementary material

Supplementary Information
