# Supplementary material for: Geometric metasurface enabling polarization independent beam splitting
Source: Sci Rep. 2018 Jun 21;8:9468. doi: 10.1038/s41598-018-27876-2 (PMC6013463; doi:10.1038/s41598-018-27876-2)
Supplement: Supplementary file 1 — Supplementary Information [file 41598_2018_27876_MOESM1_ESM.docx]

**SUPPLEMENTARY INFORMATION**

**Geometric metasurface enabling polarization independent beam splitting**

Gwanho Yoon^1^, Dasol Lee^1^, Ki Tae Nam^2^ and Junsuk Rho^1,3,4^*

^1^Department of Mechanical Engineering, Pohang University of Science and Technology (POSTECH), Pohang 37673, Republic of Korea

^2^Department of Materials Science and Engineering, Seoul National University, Seoul 08826, Republic of Korea

^3^Department of Chemical Engineering, Pohang University of Science and Technology (POSTECH), Pohang 37673, Republic of Korea

^4^National Institute of Nanomaterials Technology (NINT), Pohang 37673, Republic of Korea

*[jsrho@postech.ac.kr](mailto:jsrho@postech.ac.kr)


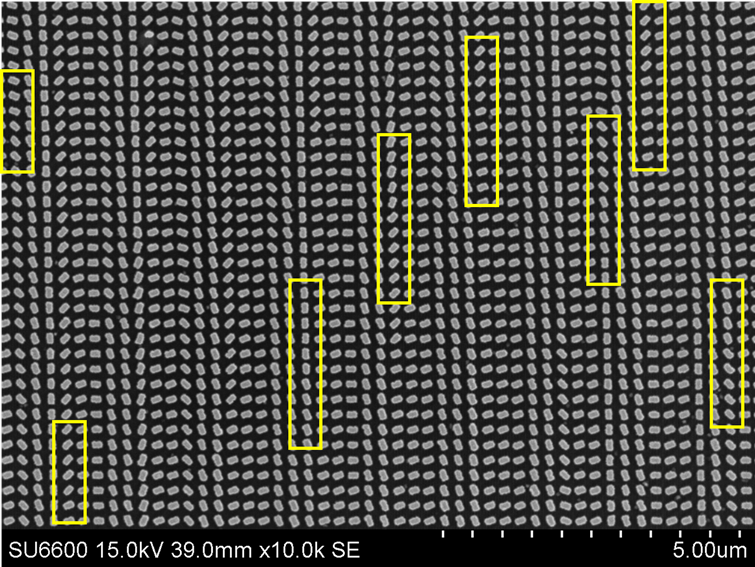


**Figure S1.** SEM image of the fabricated device. The yellow boxes show the inconsistent phase profile along the vertical direction, which comes from the GS algorithm with the random phase mask.


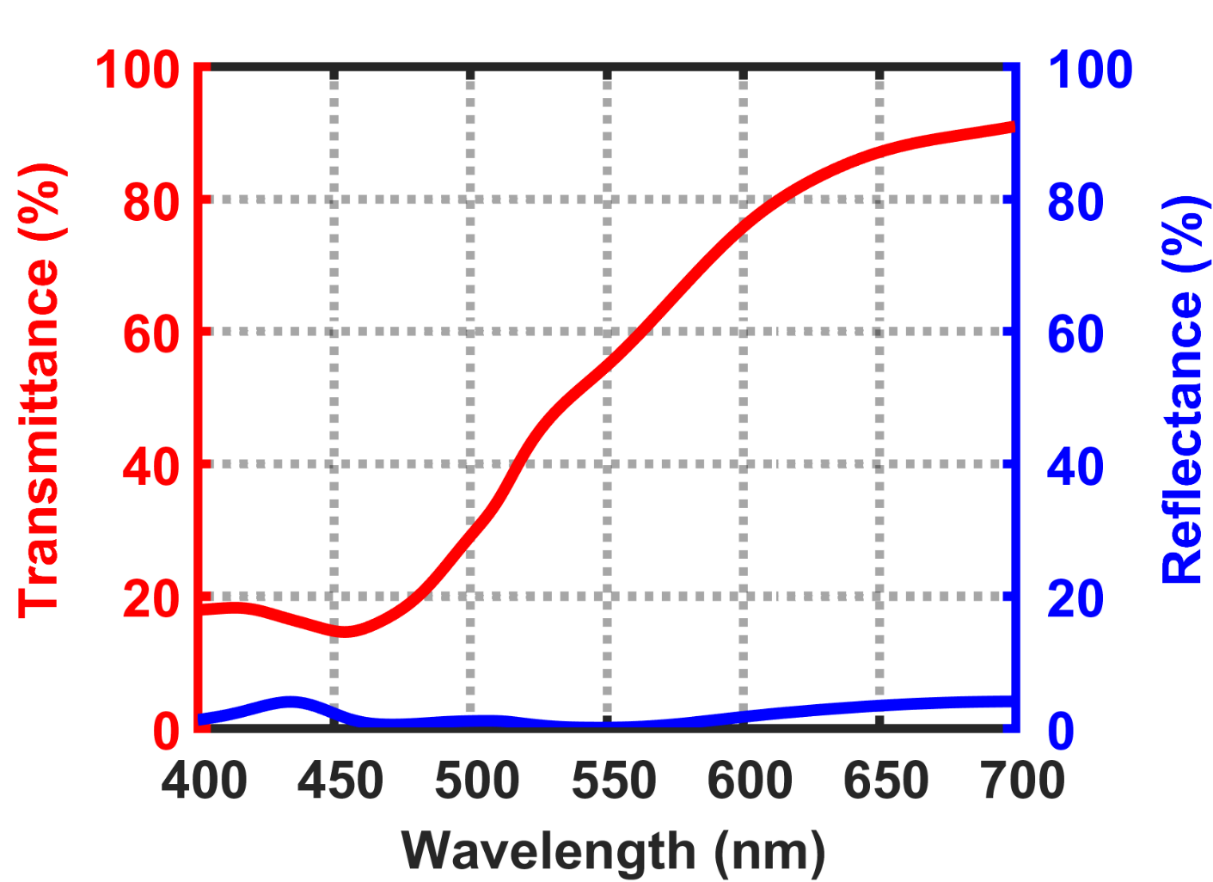


**Figure S2.** Calculated transmittance and reflectance spectrum of the unit cell for circularly polarized light incidence. The nanostructure is designed to maximize the CPT as well as minimize reflectance.
